# The Relationship between Subjective Risk Intelligence and Courage with Working Performance: The Potential Mediating Effect of Workplace Social Courage

**DOI:** 10.3390/ejihpe12040031

**Published:** 2022-04-07

**Authors:** Paola Magnano, Giuseppe Santisi, Andrea Zammitti, Rita Zarbo, Vittorio Edoardo Scuderi, Giusy Danila Valenti, Palmira Faraci

**Affiliations:** 1 Faculty of Human and Social Sciences, Kore University, 94100 Enna, Italy; rita.zarbo@unikore.it (R.Z.); palmira.faraci@unikore.it (P.F.); 2 Department of Science of Education, University of Catania, 95121 Catania, Italy; gsantisi@unict.it (G.S.); andrea.zammitti@phd.unict.it (A.Z.); 3 Department of Management, Kingston University, London KT1 2EE, UK; v.scuderi@kingston.ac.uk; 4 Department of Psychological, Pedagogical, Physical Exercise and Educational Sciences, University of Palermo, 90133 Palermo, Italy; giusydanila.valenti@unipa.it

**Keywords:** courage, workplace social courage, risk, uncertainty, workers, risk intelligence

## Abstract

Background: There is a growing attention toward the construct of courage from a psychological point of view; recently, courage has been related with numerous positive individual behaviors and outcomes, such as coping strategies and subjective wellbeing, and an increasing number of studies explore the role of courage in the working and organizational environments. The present study is aimed to analyze the effect that individual courage—together with risk intelligence—and workplace social courage have on working performance; Methods: The participants are 961 Italian workers, balanced by gender; the measures used are: Courage, Subjective Risk Intelligence Scale, Workplace Social Courage Scale, and Performance Scale. Data were analyzed using Structural Equation Models; Results: The results show the effect of subjective risk intelligence and courage on working performance, both directly and through the mediation of workplace social courage; Conclusions: Suggestions for further research and practical implications are discussed.

## 1. Introduction

In times of risk and uncertainty, the unpredictability in the social and economic environment, including the workplace, requires the activation of positive resources that enable the individuals to cope with difficulties and unexpected circumstances. At the beginning of the third millennium, Positive Organizational Behavior (POB) [1] applied the principles of the emerging positive psychology movement [2] to the organizational behavior field. Although positive psychology was, principally, focused on clinical applications, Luthans and Avolio [3] felt that it was important to deepen how such a positive focus could provide a new perspective in studying the work settings. POB is defined as “the study and application of positively oriented human resource strengths and psychological capacities that can be measured, developed, and effectively managed for performance improvement in today’s workplace” [1] (p. 59) and considers the positive state-like psychological resource capacities open to development; it also includes positive behaviors (e.g., organizational citizenship and courageous principled action) and positive organizations [3]. POB focuses on the examination of the workers’ strengths and psychological capacities for development and performance improvement. The strengths considered in this theoretical and methodological framework (encompassed in the construct of psychological capital) are self-efficacy, hope, resilience, and optimism; they have been found to be related to various employees’ attitudes, behaviors, and performance, such as job satisfaction, and, with negative relationships, turnover, or quitting a job [4,5]; moreover, positive workers’ resources have positive effects on organizational citizenship behaviors—such as helping a coworker and/or supporting the organization—and negative effects on counterproductive work behaviors [6]. Finally, regarding performance at the workplace, higher levels of psychological capital are positively associated with better working performance [6].

Besides these aforementioned constructs, Luthans and Avolio [5] suggested other positive concepts to be included in POB, comprising, among others, courage. Following these suggestions, we have hypothesized that individual and workplace courage could positively affect working performance. However, psychological research has only recently given attention to courage; in the past, courage was regarded as a virtue; in the last decades, some researchers have contributed to construct a socially relevant view of courage in the experience of daily life and the mind of every person [7]. Moreover, Howard et al. [8] underlined that, regarding the role of courage in working environments, it was considered an important resource limited to highly stressful and difficult occupations, and, only recently, its role in positively influencing the usual working interactions has been explored: recent studies highlighted its positive effect on coping strategies [9] and quality of life [10], its relationship with psychological capital [11] and organizational citizenship behaviors [12], and the related ethical decision-making [13].

Courage has been found to support individuals to deal with external problems by overcoming the fear of doing the things [14].

In a world characterized by transitions and insecurity, in which prominent features are risk, uncertainty, frequent changes and transitions in the working experience, and the instability of future perspectives, intelligent risk management and courage can be positive resources to deal with feelings of fear and to respond effectively to demanding work (and life) contexts.

## 2. Literature Review

### 2.1. Relationship between Subjective Risk Intelligence and Workplace Social Courage

Subjective risk intelligence can be defined “as the capacity of a person to effectively assess the pros and cons of a decision in situations in which not all outcomes are totally expected” [15] (p. 968); individuals with high level of subjective risk intelligence are able to estimate the risks effectively, evaluating their advantages and disadvantages as well as approaching them as an opportunity rather than like a threat, thus feeling able to manage the lack of information, or uncertainty, as an opportunity. In this construct, the term *risk* is considered as an adaptive dimension, which allows one to focus not only on the negative aspects of a risky opportunity, but also to assess its potential benefits [16,17]. The conceptualization of subjective risk intelligence [15,17] provides a four-dimensional construct, composed of the following dimensions: (1) imaginative capability, defined as the generation of novel and potentially useful ideas, such as initiative taking and originality; (2) problem solving self-efficacy, which encompasses self-confidence and belief in one’s capacity to handle situations, including the ability to make decisions; (3) emotional stress management, which is the capacity to modulate emotional responses in stressful situations; and (4) positive attitude toward uncertainty, the ability to attribute positive significance to uncertainty, perceiving it as an opportunity rather than a threat.

Risk intelligence seems closely related to courage, and the riskiness of action is included in several definitions of courage [18]. Shelp [19] (p. 343) defined courage as “the disposition to voluntarily act, perhaps fearfully, in a dangerous circumstance, where the relevant risks are reasonably appraised, in an effort to obtain or preserve some perceived good for oneself or others, recognizing that the desired perceived good may not be realized”; Klein and Napier [20] identified five factors in courage: candor, purpose, rigor, risk, and will; Goud [21] defined courage as actions in front of the risk; finally, Norton and Weiss [22] define courage as persistence despite fear. Much of the literature on this topic [21,23,24,25,26,27] is convinced that a courageous act is made up of these three components: (1) a morally worthy goal, (2) intentional action, and (3) the perception of risk, threat, or obstacle. The component of risk in the definition of courage seems intrinsically linked [28]. According to some authors [19,23,26,29,30], fear and courage are connected. However, fear drives people to avoid risks, and fear in the workplace has become so widespread that avoiding risks seems normal [31].

Linked to the construct of courage, it is possible to find the constructs of social courage and workplace courage. Social courage implies that the actors of a courageous behavior could risk damaging their social image [8]. Workplace courage includes three important elements: (1) the fact that it is something pertinent to the workplace, (2) the riskiness, and (3) the worthiness of an act [18].

Organizations are risky places for their workers, due to their formal or informal hierarchies and the quality of the relationships or the behaviors required; they are a source of physical risks (such as bodily injuries), social risks (such as the loss of friends), psychological risks (anxiety), and economic risks (such as the loss of career) [30,32,33]. For these reasons, courage is important in organizations, as it can represent a psychological and behavioral resource to overcome risks and the subsequent fear [34]. Several researchers [35] identified courage as a core characteristic of organizations and a necessary component to be a leader [36,37,38,39]. Courage in organizations allows long-term goals to be set, without being paralyzed by fear [40]. Courageous leaders gain benefits for themselves, their followers, and the whole organization [41].

### 2.2. Relationships between Subjective Risk Intelligence and Working Performance

As highlighted by Craparo and colleagues [15], the concept of risk intelligence has its origin in the managerial sector and in Apgar’s [42] definition of risk intelligence as the ability, at the individual or organizational level, to estimate risks effectively. More recently, Evans [16] provided a broader definition, which includes the ability to accurately estimate the probability of an event; they propose that some individuals are more able than others to evaluate the risks involved in making a decision and to handle situations of uncertainty, overcoming feelings of anxiety and uneasiness, which are paralyzing for others. Accordingly, in the subsequent theorizations [15], it is characterized by a positive attitude toward uncertainty, self-efficacy, imaginative capability, and stress management. As underlined by the authors, subjective risk intelligence helps individuals in evaluating uncertainty as an opportunity rather than a threat, in acting creatively, in managing emotional stress, and by having high levels of perceived self-efficacy in making decisions.

Subjective risk intelligence has been found to play a role in career transition processes [43]; in self-employment and entrepreneurship propensity; and in career adaptability [44], job satisfaction, and career success [15]. There are not studies that directly explored the relationship between subjective risk intelligence and working performance, but based on the relationships with other positive working and career-related outcomes, we can hypothesize that high levels of subjective risk intelligence, characterized by the ability to effectively manage an uncertain situation without being overcome by feelings of anxiety, by using creativity to deal with difficulties, could be a positive predictor of a good performance in working contexts.

### 2.3. Courage, Workplace Social Courage and Working Performance

As previously defined, courage is conceptualized as persistence despite fear [22]; so, courage can be described as “a behavior characterized by the perpetuation of effort despite the subjective feeling of fear” [45] (p. 3). Some recent studies have related courage to several positive adaptive outcomes and career-related variables [46], including positive work behavior outcomes [45] and entrepreneurial success [47]. There is little evidence on the relationships between courage and other working and organizational variables, such as employability and meaningful work [10] as well as psychological capital [11]. In our literature review, we found only a few studies that have investigated the relationship between courage and working performance [48].

From a psychological perspective, courage has been conceptualized differently according to the domains it is applied to or the behaviors it requires: most authors consider types of courage to be differentiated by the risks (i.e., physical courage, social courage) or goals (i.e., moral courage) involved in a behavior [49]. Categorization according to the risks allows for the identification of three types of courageous behaviors that may be important to workplace interactions: physical, moral, and social courage [8,50]. According to the definition provided by Howard and Cogswell [49] (p. 3), “social courage is an intentional, deliberate, and prosocial behavior in which the risks involved are to the actor’s esteem in the eyes of others [8]”; social courage has been suggested to be the most important dimension of courage to the modern workplace [8,27,34,51], significantly related to workplace outcomes [27,51,52]; and Howard et al. [8] highlighted that workers with social courage tend to show the necessary behaviors to achieve these working outcomes. Specifically, socially courageous behaviors could enhance performance. Tkachenko et al. [53] have shown how relevant behavioral courage could be to performance within the workplace; in fact, the study presented behavioral courage as expressing its role as a predictor of job performance. Additional evidence from Palanski et al. [54] has found similar results, finding that the behavioral courage of managers is positively correlated with performance.

To date, little is known about the relationships between the dimensions presented, especially the role of courage and workplace social courage on working performance. The present study aims to fill this gap.

## 3. Aims of the Research

The analysis of the existing literature presented above provides useful insights to the rationale underlying our research purposes. Subjective risk intelligence helps individuals to deal effectively with the risks; this construct seems closely related to courage, as both include facing risky situations or perform risky actions [18]; workplace social courage, that is a type of courage that involves prosocial behaviors in which one of the risks involved is ‘losing face’ in the eyes of others [8], can support the management of risks in the workplace, enhancing working performance [8,53,54]. So, the aim of the present study is exploring the relationships that risk intelligence, courage, and workplace social courage have with working performance. In detail, we hypothesize the following:

**Hypothesis** **1** **(H1):** *Subjective riskintelligence affects directly working performance*.

**Hypothesis** **2** **(H2):** *Subjective risk intelligence affects working performance through the mediation of workplace social courage*.

**Hypothesis** **3** **(H3):** *Courage affects working performance*.

**Hypothesis** **4** **(H4):** *Courage affects working performancethrough the mediation of workplace social courage*.

**Figure 1:**
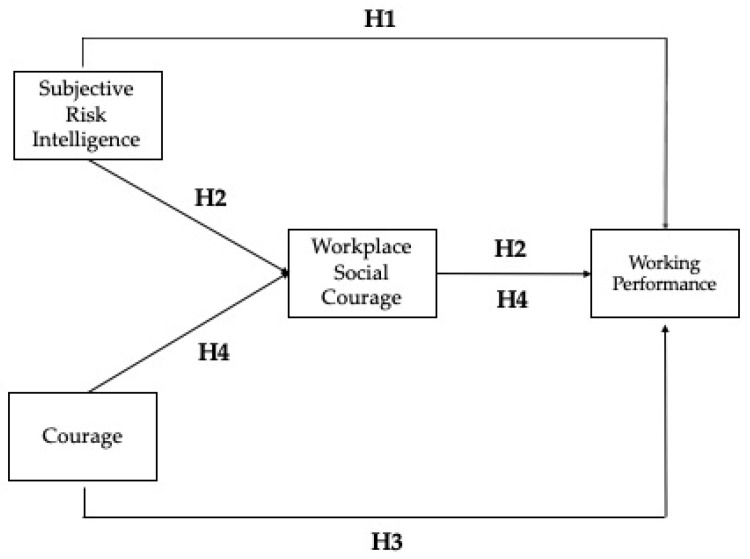
The conceptual model.

The conceptual model is reported in Figure 1.

## 4. Procedure

### 4.1. Participants

The participants were 961 Italian workers (male = 455, 47.3%; female = 501, 52.1%; n.r. = 5, 0.5%) aged between 18 and 70 years (M = 40.72; SD = 12.89).

The participants were recruited from the population of workers in different types of organizations, coming from different Italian regions, through a convenience sample. Most of them had a high school degree (471, 49%) or a university degree (381, 39.6%); the remaining part had a middle school degree (98, 10.2%); and a very small part did not respond (11; 1.1%).

They work in public (404, 42%), private (495, 51.5%) and nonprofit (51, 5.3%) organizations (n.r. = 11; 1.1%).

The respondents participated voluntarily, and they were free to abandon their participation at any time during the administration. Data were collected through an online survey, carried out by previously trained researcher assistants. The survey was accompanied by the following indications: “Dear participant, The Workplace Courage research project involves the participation of workers and aims to analyze and understand the factors that can influence work performance (such as courage and risk intelligence). You will be asked to answer questions that identify typical working situations or personal situations, marking an answer among the alternatives proposed. There are no right or wrong answers but we are interested in your point of view. The data will be processed in aggregate form and will not be possible to trace the characteristics of the individual participant, the absolute anonymity of the persons involved is guaranteed, in full compliance with the laws on the protection of privacy. Therefore, please answer all questions as sincerely as possible. Thank you for your cooperation. Good job!” The first page contained the contacts of the research proponents. There were no monetary incentives for administration and participation.

The following inclusion criteria were considered: minimum age of 16 (this is the age from which in Italy compulsory education has ended, and people can access work); being workers; being a resident and working in Italy. In addition to requesting age, to prevent unemployed people from being mistakenly included in the sample, the survey asked to indicate the employment status. In addition, it was required to indicate the region of residence, and in which region one carries out one’s professional activity. No exclusion criteria were considered besides the lack of inclusion requirements.

The respondents provided an individual code composed of the first three letters of the first name and the first three letters of the last name, which, when matched with other demographic information, was permitted to check eventual double compilation; the survey was approved by the ethical commission of the universities involved, and the research followed the ethical rules of the Italian Psychological Association.

The sample size was determined using G*Power software version 3.1.9.7 [55,56]. The parameters used were the following: Effect size f2 = 0.15; α err prob = 0.05; power = 0.95; number of predictors = 3, as indicated in the literature [57]. The minimum sample size for performing Pearson’s correlation coefficient was found to be 115; the minimum sample size for performing multiple linear regression was 119, which is lower than this study’s actual sample size. Based on the priori analyses, our sample is appropriate for testing the proposed model [58].

### 4.2. Measures

All the measures used are previously validated instruments. Thus, the CFA of the measures, reported below, confirms their psychometric properties on the sample of the study.

#### 4.2.1. Subjective Risk Intelligence Scale

The Subjective Risk Intelligence Scale (SRIS) [15] is composed of 21 items with a 5-point Likert scale, from 1 (totally disagree) to 5 (totally agree); sample item: Totally new situations scare me. The Cronbach’s alpha calculated on the study sample was 0.88.

#### 4.2.2. Courage Measure

The Italian adaptation of the Courage Measure (CM) [22,45] is a six-item scale adapted from the short version of CM proposed by Howard and Alipour [59]; responses are provided on a 7-point Likert scale from 1 (never) to 7 (always); sample item: I will do things even though they seem to be dangerous. The Cronbach’s alpha of the study sample was 0.85.

#### 4.2.3. Workplace Social Courage Scale

The Workplace Social Courage Scale (WSCS) [8] is composed of 11 items with a 7-point Likert scale, from 1 (totally disagree) to 7 (totally agree); sample item: Although it may damage our friendship, I would tell my superior when a co-worker is doing something incorrectly. The Cronbach’s alpha of the study sample was 0.76.

#### 4.2.4. Working Performance

Working Performance was measured with a four-item scale [60] answered on a 5-point Likert scale, from 1 (very bad) to 5 (very good). The Cronbach’s alpha calculated on the sample of the study is 0.76.

## 5. Data Analyses

To conduct data analysis, we used Linear structural equation models (Lisrel 8.80). Firstly, we tested the measurement model, performing a confirmatory factor analysis (CFA). Then, we estimated the structural equation model. In evaluating the adequacy of the data to the model, we used the following goodness-of-fit indices: chi-square (χ^2^), the χ^2^/df ratio, the comparative fit index (CFI), and the root mean square error of approximation (RMSEA). For the CFI, values over 0.90 suggest an acceptable fit, while values over 0.95 suggest a good fit. According to Browne and Cudeck [61], a RMSEA < 0.09 is still an acceptable threshold in smaller samples. To estimate convergent validity, we used composite reliability (CR; the degree to which the scale indicators reflect an underlying factor [62]; acceptable threshold > 0.70) and the average variance extracted (AVE; the average percentage of variation explained among the items of a construct [63]; acceptable threshold > 0.50).

Secondly, we verified the mediation hypothesis using Jamovi v.2.2.1, which provides the significance of the indirect effects using the bootstrapping method, with 5000 repetitions and establishing a confidence interval (CI) of 95%.

Finally, mean, standard deviations and correlations were obtained using SPSS 25.0. Missing values for the relevant items were estimated using the expectation maximization method. None of the items had more than 5% missing values, indicating the appropriateness of this option.

## 6. Results

### 6.1. Descriptive Statistics and Correlations

Table 1 shows the mean and standard deviation of the measures (SRIS, Courage, WSCS, and Performance), and the correlations between the variables were calculated through the Pearson’s r.

### 6.2. CFA of the Measures

Before testing the structural model, an assessment of the properties of the measurement model is required. We conducted a Confirmatory Factorial Analysis (CFA), using Lisrel 8.80, according to Harman’s single-factor test, to diagnose if common-method variance was a problem. A comparison between the hypothesized model and a model with one factor, in which all the items loaded on a unique factor, revealed that the four-factors\ model provided a better fit for the data in all the CFA indexes of fit (four-factor model: χ^2^(813) = 4632.847; CFI = 0.92; GFI = 0.75; RMSEA = 0.08; SRMR = 0.07; AIC = 6808.161;1-factor model: χ^2^(819) = 7197.309; CFI = 0.86; GFI = 0.66; RMSEA = 0.11; SRMR = 0.09, AIC = 10724.221). The differences were significant, according to a comparison of the models’ χ^2^ values and degrees of freedom: Δχ^2^(6) = 2564.462 (*p* < 0.001). According to these results, the common-method bias could be considered overcome.

All the items loaded significantly on the hypothesized latent variables (factor loadings range 0.30–0.80; t > 2.58), indicating convergent validity. The values of composite reliability (CR > 0.75) are good for all the constructs, indicating that all the items constantly measure the same construct; the values of average variance extracted (AVE > 0.40) indicate an acceptable items’ discriminant capacity.

### 6.3. Structural Model and Mediational Analysis

**Figure 2:**
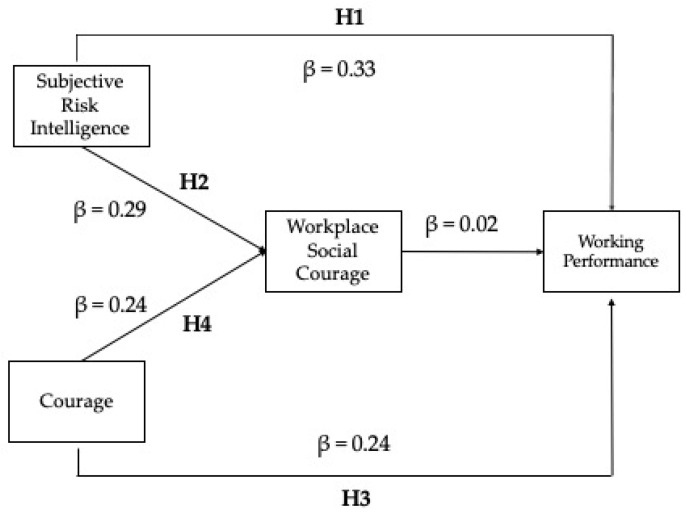
The final model.

We tested our hypotheses using structural equation modeling analysis. The main fit indices suggest acceptably fit indexes (χ^2^(813) = 4632.847; CFI = 0.92; RMSEA = 0.08; SRMR = 0.07). The final model is presented in Figure 1, reporting the standardized β to indicate the relationships between the variables. As represented in Figure 2, subjective risk intelligence: (1) shows a direct relationship with the workplace social courage; (2) has a direct relationship with performance; courage, similarly, is directly related with the workplace social courage and with performance; workplace social courage has a direct relationship with performance.

We tested the mediational hypothesis through the estimation of the significance of the indirect effects, using the bootstrapping method. The results of the mediations are reported presenting the standardized β and the confidence intervals (C.I.) as 95%, which indicate the significance of the effect with a 5% of probability of error (C.I. that do not comprise 0 are significant). The results, reported in Table 2, show that the path from subjective risk intelligence to workplace social courage is significant (β = 0.29, *p* < 0.001), as well as the path from workplace social courage to performance (β = 0.09, *p* = 0.012), supporting Hypotheses 1 and 2; moreover, the path from courage to workplace social courage is significant (β = 0.24, *p* < 0.001), as well as the path from workplace social courage to performance (β = 0.09, *p* = 0.012), showing a direct and indirect effect of courage on performance, mediated by workplace social courage. These results support Hypotheses 3 and 4.

## 7. Discussion

The aim of the present study was to verify the relationships that subjective risk intelligence and courage have with working performance, analyzing the role played by workplace social courage. Data analyses support all the hypotheses. So, subjective risk intelligence and courage have a direct effect on working performance, and these relationships are partially mediated by workplace social courage.

Although there are no other studies that directly explore the relationship between subjective risk intelligence and working performance, our results can be read starting from the definition of subjective risk intelligence and its dimensions. In fact, following the conceptualization of subjective risk intelligence [15], individuals with a high level of risk intelligence are more ready to interpret the changing situations, the lack of information, or the uncertainty, as a chance rather than a threat, especially when a decision is necessary; on the contrary, low levels of subjective risk intelligence are related to the difficulty in making decisions when the results are not predictable, which can lead to serious distress or impairment in interpersonal, occupational, or other areas of social functioning. This individual psychological resource has recently been related to career expectations [43] and can play a role in achieving positive outcomes in social functioning, both in personal and professional situations. Therefore, according to the four dimensions that compose the construct of subjective risk intelligence (attitude toward uncertainty, imaginative capability, problem solving self-efficacy, and stress management), workers high in risk intelligence are creative, have good levels of self-efficacy in problem solving, have a positive attitude toward uncertainty, and manage stressing situations without being overcome by them. Each of these characteristics has been found to be related to working performance. With regard to the first dimension, numerous studies have associated creativity with performance, both in academic contexts [64,65] and in working contexts [66,67,68], demonstrating that performance could be enhanced (and intentions to quit lowered) if the employees were enabled to use their creativity in managing their job. More recently, the American Psychological Association [69] underlined that creativity, along with cognitive ability, leadership, integrity, attendance, and cooperation, are the factors which predict job performance; finally, Yee et al. [70] demonstrated the importance of the psychological climate for creativity as a predictor of the work performance.

The second dimension, self-efficacy, is considered the strongest predictor of performance in many domains [71,72,73]; as underlined by Sitzmann and Yeo [74], a massive amount of research has found positive relationships between self-efficacy and performance, both in academic [75] and working contexts. Self-efficacy has been shown to increase performance stronger than goal setting, feedback interventions, or behavior modifications [76,77]. Moreover, a great number of studies have found positive correlations between self-efficacy and performance at the within-person level of analysis [78,79].

The third dimension, positive attitude toward uncertainty, has been related to a better performance in previous studies: i.e., Kornilov et al. [80] found that tolerance for uncertainty is a predictor of a better performance in decision-making; in their review, Furnham and Marks [81] highlighted that tolerance for uncertainty has been related to several behavioral outcomes, including managerial performance [82]. Finally, Chatterjee and Das [83] proposed a theoretical framework to predict entrepreneurial success, which included tolerance for ambiguity as a key psychological factor.

The fourth dimension of subjective risk intelligence is referred to as the ability to manage stressful situations without being overcome by feelings of anxiety; the inverted-u relationship between anxiety and performance is well known and largely documented in psychology. Recent studies have proven the association with effective stress management and performance, in academic contexts [84,85], and in different working contexts [86,87,88,89].

Analyzing the role of courage, it is related, both directly and indirectly, to working performance, through the partial mediation of workplace courage. Courage has been included in positive psychological strength, meeting the Positive Organizational Behavior criteria [4], and has an impact on performance [5]. Moreover, courage is already comprised of the positive emotions [90] that are found to be related to job performance [91].

Workplace social courage, in our study, has the role of mediator between subjective risk intelligence and individual courage with working performance. Many recent studies have demonstrated the mediational role of courage between individual psychological resources and various outcomes [9,10,17,46]; the workplace social courage, in this study, plays a similar role, mediating between individual psychological resources (subjective risk intelligence and individual courage) and outcome (working performance). The relationship between workplace social courage and performance has been hypothesized in previous studies but not clearly explained by empirical results, yet. Using the words of Howard and colleagues [8] (p. 676) “[…] qualitative research supports that social courage is linked to negative feedback giving, leading others effectively, organizational citizenship behaviors, and many other workplace outcomes [27,51,52] […]. Those with social courage […] may be more likely to perform the required behaviors to achieve these outcomes. In turn, these behaviors improve performance […]”. Therefore, our study provides the first empirical demonstration of the relationships among workplace social courage and working performance. Risk-intelligent and courageous workers tend to activate, more likely, socially courageous behaviors in the workplace, such as confronting coworkers’ problematic behaviors or situations that could damage the actor’s social image, which, in turn, have a positive effect on working performance.

Reading the findings of the study in the framework of POB, our study provides new empirical evidences that courage, specifically social courage, can play a role in POB, in line with the results of a recent study conducted by Dhir and Sharma [92]: they proposed expanded psychological capital, introducing courage among the other positive strengths; in fact, their research, using a qualitative method, showed that courage was identified as a prerequisite to work-related actions, and as a reliable psychological resource for positive energy for the individuals that helped to improve their performance.

## 8. Conclusions and Limitations

The results of this study provide a small but significant contribution to the research on risk intelligence and workplace social courage. Regarding the first construct, there is growing attention toward the construct of subjective risk intelligence, and this is the first study that explores the relationship between subjective risk intelligence and working outcomes, specifically working performance; regarding workplace social courage, following Howard and colleagues’ [8] suggestion to demonstrate the influence of social courage on performance outcomes, we found the mediational role of this construct between psychological resources and working performance. So, the present study provides a new piece of knowledge about the construct of courage applied to the working domain, which could contribute to extending the conceptualization of these constructs and to identifying the role they play in the working and organizational behaviors.

Moreover, the results of our study have both theoretical and practical implications.

From a theoretical point of view, among the positive concepts that can be counted in POB, in addition to courage, as already suggested by Luthans and Avolio [3], our study also suggests to count risk intelligence as a useful resource in working behaviors.

From a practical point of view, in considering the changing and uncertain nature of the current labour market, this research shows the importance of taking into account and activating, for the benefit of companies and workers, actions aimed at the acquisition or enhancement of positive personal resources, such as courage and risk intelligence, even in the workplace.

However, the study has some limitations: first of all, it is a cross-sectional study, so we cannot definitely conclude about the causal relationships between the variables analyzed; secondly, the convenience sampling cannot allow to generalize the results to the workers’ population; and thirdly, performance is detected through a self-report measure and not with an objective one.

In conclusion, further research, firstly, could explore the role of courage and workplace social courage on other organizational dimensions that require the workers to courageously act, putting at risk their image, role, or their reputation on the workplace; for example, prosocial rule breaking behaviors, exploring how the psychosocial climate [93] and diversity climate [94] could favor or interfere in such types of behaviors.

## Figures and Tables

**Table 1:** Descriptive statistics and correlations between the variables.

	M	SD	1	2	3	4
SRI	3.65	0.57	−			
Courage	5.24	1.17	0.52 ***	−		
WSC	5.05	0.85	0.42 ***	0.39 ***	−	
Working performance	4.15	0.53	0.49 ***	0.43 ***	0.31 ***	−

Note. SRI = subjective risk intelligence; WSC = workplace social courage; *** *p* < 0.001.

**Table 2:** Effects of subjective risk intelligence and courage on working performance through workplace courage (standardized β).

Paths	Indirect Effect		Direct Effect		Total Effect	
	β	C.I. 95%	β	C.I. 95%	β	C.I. 95%
Subjective risk intelligence—Workplace courage—Working performance	0.02	0.004–0.04	0.33	0.24–0.38	0.36	0.28–0.39
Courage—Workplace courage—Working performance	0.02	0.004–0.02	0.22	0.07–0.13	0.24	0.08–0.14

## Data Availability

The data that support the findings of this study and the scales used are available on osf.io/rvkw2 (accessed on 5 March 2020).

## References

[1] Luthans F. (2002). The need for and the meaning of positive organizational behavior. J. Organ. Behav..

[2] Seligman M.E.P., Csikszentmihalyi M. (2000). Positive psychology. Am. Psychol..

[3] Luthans F., Avolio B. (2009). The “point” of positive organizational behavior. J. Organ. Behav..

[4] Luthans F., Avolio B.J., Avey J.B., Norman S. (2007). Positive psychological capital: Measurement and relationship with performance and satisfaction. Pers. Psychol..

[5] Luthans F., Norman S.M., Avolio B.J., Avey J.B. (2008). The mediating role of psychological capital in the supportive organizational climate–employee performance relationship. J. Organ. Behav..

[6] Avey J.B., Reichard R.J., Luthans F., Mhatre K.H. (2011). Meta-analysis of the impact of positive psychological capital on employee attitudes, behaviors, and performance. Hum. Resour. Dev. Q..

[7] Lopez S.J., O’Byrne K.K., Petersen S., Lopez S.J., Snyder C.R. (2003). Profiling courage. Positive Psychological Assessment: A Handbook of Models and Measures.

[8] Howard M.C., Farr J.L., Grandey A.A., Gutworth M.B. (2016). The creation of the workplace social courage scale (WSCS): An investigation of internal consistency, psychometric properties, validity, and utility. J. Bus. Psychol..

[9] Magnano P., Paolillo A., Platania S., Santisi G. (2017). Courage as a potential mediator between personality and coping. Personal. Individ. Differ..

[10] Magnano P., Santisi G., Zammitti A., Zarbo R., Di Nuovo S. (2019). Self-perceived employability and meaningful work: The mediating role of courage on quality of life. Sustainability.

[11] Santisi G., Lodi E., Magnano P., Zarbo R., Zammitti A. (2020). Relationship between psychological capital and quality of life: The role of courage. Sustainability.

[12] Hannah S.T., Avolio B.J., Walumbwa F.O. (2011). Relationships between authentic leadership, moral courage, and ethical and pro-social behaviors. Bus. Ethics. Q..

[13] Ayling D. Fostering moral courage: What do business students learn about professional ethics in cooperative education placements?. Proceedings of the Conference Proceedings: New Zealand Association for Cooperative Education Annual Conference.

[14] Sosik J.J., Gentry W.A., Chun J.U. (2012). The value of virtue in the upper echelons: A multisource examination of executive character strengths and performance. Leadersh. Q..

[15] Craparo G., Magnano P., Paolillo A., Costantino V. (2018). The Subjective Risk Intelligence scale. The development of a new scale to measure a new construct. Curr. Psychol..

[16] Evans D., Roeser S., Hillerbrand R., Sandin P., Peterson M. (2012). Risk Intelligence. Handbook of Risk Theory.

[17] Magnano P., Guarnera M., Buccheri S.L., Zarbo R., Craparo G. (2021). Adaptation and Validation of the Subjective Risk Intelligence Scale for Italian Adolescents (SRIS-A). Child Psychiatry Hum. Dev..

[18] Detert J.R., Bruno E.A. (2017). Workplace courage: Review, synthesis, and future agenda for a complex construct. Acad. Manag. Ann..

[19] Shelp E.E. (1984). Courage: A neglected virtue in the patient-physician relationship. Soc. Sci. Med..

[20] Klein M., Napier R. (2003). The Courage to Act: 5 Factors of Courage to Transform Business.

[21] Goud N.H. (2005). Courage: Its Nature and Development. J. Humanist. Couns. Educ. Dev..

[22] Norton P.J., Weiss B.J. (2009). The role of courage on behavioral approach in a fear-eliciting situation: A proof-of-concept pilot study. J. Anxiety Disord..

[23] Kilmann R., O’Hara L., Strauss J., Burke R.J., Cooper C.L. (2013). Developing and validating a quantitative measure of organizational courage. Voice and Whistleblowing in Organizations.

[24] O’Byrne K.K., Lopez S.J., Peterson S. Building a theory of courage: A precursor to change?. Proceedings of the 108th Annual Convention of the American Psychological Association.

[25] Rate C.R., Clarke J.A., Lindsay D.R., Sternberg R.J. (2007). Implicit theories of courage. J. Posit. Psychol..

[26] Woodard C.R. (2004). Hardiness and the Concept of Courage. Consult. Psychol. J. Pract. Res..

[27] Worline M.C., Wrzesniewski A., Rafaeli A., Lord R., Klimoski R., Kanfer R. (2002). Courage and work: Breaking routines to improve performance. Emotions at Work.

[28] Magnano P., Lodi E., Zammitti A., Patrizi P. (2021). Courage, career adaptability, and readiness as resources to improve well-being during the University-to-Work Transition in Italy. Int. J. Environ. Res. Public Health.

[29] Rachman S.J. (2004). Fear of contamination. Behav. Res. Ther..

[30] Woodard C.R., Pury C.L.S. (2007). The construct of courage: Categorization and measurement. Consult. Psychol. J. Pract. Res..

[31] Warrell M. (2000). Find Your Courage.

[32] May R. (1994). The Courage to Create.

[33] Schilpzand P. (2008). Personal Courage: A Measure Creation Study.

[34] Koerner M.M. (2014). Courage as identity work: Accounts of workplace courage. Acad. Manag. J..

[35] Cavanagh G.F., Moberg D.J. (1999). The virtue of courage within the organization. Res. Ethical Issues Organ..

[36] Kouzes J.M., Posner B.Z. (2008). A Leader’s Legacy.

[37] Lombardo M.M., Eichinger R.W. (2003). For Your Improvement: A Development and Coaching Guide.

[38] Spreitzer G.M., McCall M.W., Mahoney J.D. (1997). Early identification of international executive potential. J. Appl. Psychol..

[39] Teal T.A. (1996). Tales of Management Courage and Tenacity.

[40] Sarros J., Cooper B. (2006). Building character: A leadership essential. J. Bus. Psychol..

[41] Dvir T., Shamir B. (2003). Follower developmental characteristics as predicting transformational leadership: A longitudinal field study. Leadersh. Q..

[42] Apgar D. (2006). Risk Intelligence: Learning to Manage What We Don’t Know.

[43] Lodi E., Zammitti A., Magnano P. (2021). Risk intelligence as a resource in career transition: The role of college satisfaction on the visions about future jobs. Eur. J. Investig. Health Psychol. Educ..

[44] Savickas M.L., Porfeli E.J. (2012). Career adapt-abilities scale: Construction, reliability, and measurement equivalence across 13 countries. J. Vocat. Behav..

[45] Ginevra M.C., Santilli S., Camussi E., Magnano P., Capozza D., Nota L. (2019). The Italian adaptation of courage measure. Int. J. Educ. Vocat Guid..

[46] Ginevra M.C., Magnano P., Lodi E., Annovazzi C., Camussi E., Patrizi P., Nota L. (2018). The role of career adaptability and courage on life satisfaction in adolescence. J. Adolesc..

[47] Bockorny K.M. (2015). Psychological Capital, Courage, and Entrepreneurial Success. Ph.D. Thesis.

[48] Lindh I.B., Barbosa da Silva A., Berg A.B., Severinsson E. (2010). Courage and nursing practice: A theoretical analysis. Nurs. Ethics.

[49] Howard M.C., Cogswell J.E. (2019). The left side of courage: Three exploratory studies on the antecedents of social courage. J. Posit. Psychol..

[50] Sekerka L.E., Bagozzi R.P., Charnigo R. (2009). Facing ethical challenges in the workplace: Conceptualizing and measuring professional moral courage. J. Bus. Ethics.

[51] Geller E.S., Veazie B. (2009). The Courage Factor: Leading People-Based Culture Change.

[52] Bashir S., Khattak H.R., Hanif A., Chohan S.N. (2011). Whistle-blowing in public sector organizations: Evidence from Pakistan. Am. Rev. Public Admin..

[53] Tkachenko O., Quast L.N., Song W., Jang S. (2018). Courage in the workplace: The effects of organizational level and gender on the relationship between behavioral courage and job performance. J. Manag. Organ..

[54] Palanski M.E., Cullen K.L., Gentry W.A., Nichols C.M. (2015). Virtuous leadership: Exploring the effects of leader courage and behavioral integrity on leader performance and image. J. Bus. Ethics.

[55] Faul F., Erdfelder E., Lang A.-G., Buchner A. (2007). G*Power 3: A flexible statistical power analysis program for the social, behavioral, and biomedical sciences. Behav. Res. Methods.

[56] Faul F., Erdfelder E., Buchner A., Lang A.-G. (2009). Statistical power analyses using G*Power 3.1: Tests for correlation and regression analyses. Behav. Res. Methods.

[57] Erdfelder E., Faul F., Buchner A. (1996). GPOWER: A general power analysis program. Behav. Res. Methods Instrum. Comput..

[58] Cohen J. (1992). A power primer. Psychol. Bull..

[59] Howard M.C., Alipour K.K. (2014). Does the courage measure really measure courage? A theoretical and empirical evaluation. J. Posit. Psychol..

[60] Abramis D.J. (1994). Relationship of job stressors to job performance: Linear or an inverted-U?. Psychol. Rep..

[61] Browne M.W., Cudeck R., Bollen K.A., Long J.S. (1993). Alternative ways of assessing model fit. Testing Structural Equation Models.

[62] Fornell C., Larcker D.F. (1981). Evaluating structural equationmodels with unobservable variables and measurement error. J. Mark. Res..

[63] Hair J.F., Black W.C., Babin B.J., Anderson R.E., Tatham R.L. (1998). Multivariate Data Analysis.

[64] Chamorro-Premuzic T. (2006). Creativity versus conscientiousness: Which is a better predictor of student performance?. Appl. Cogn. Psychol..

[65] Mumford M.D., Costanza D.P., Threlfall K.V., Baughman W.A., Reiter-Palmon R. (1993). Personality variables and problem-construction activities: An exploratory investigation. Creat. Res. J..

[66] Feist G.J. (1998). A meta-analysis of personality in scientific and artistic creativity. Personal. Soc. Psychol. Rev..

[67] Feist G.J., Sterneberg G. (1999). The Influence of Personality on Artistic and Scientific Creativity. Handbook of Creativity.

[68] Oldham G.R., Cummings A. (1996). Employee creativity: Personal and contextual factors at work. Acad. Manag. J..

[69] American Psychological Association (2011). Which Traits Predict Job Performance?. http://www.apa.org/helpcenter/predict-job-performance.

[70] Yee W.F., Pink L.S., Sern M.L.C. (2014). The effect of a psychological climate for creativity on job satisfaction and work performance. Int. J. Econ. Manag..

[71] Bandura A. (1977). Self-efficacy: Toward a unifying theory of behavioral change. Psychol. Rev..

[72] Bandura A., Locke E.A. (2003). Negative self-efficacy and goal effects revisited. J. Appl. Psychol..

[73] Bandura A. (2012). On the functional properties of perceived self-efficacy revisited. J. Manag..

[74] Sitzmann T., Yeo G. (2013). A meta-analytic investigation of the within-person self-efficacy domain: Is self-efficacy a product of past performance or a driver of future performance?. Pers. Psychol..

[75] Magnano P., Lodi E., Boerchi D. (2020). The Role of Non-intellective Competences and Performance in College Satisfaction. Interchange.

[76] Stajkovic A.D., Luthans F. (1998). Self-efficacy and work-related performance: A meta-analysis. Psychol. Bull..

[77] Judge T.A., Jackson C.L., Shaw J.C., Scott B.A., Rich B.L. (2007). Self-efficacy and work-related performance: The integral role of individual differences. J. Appl. Psychol..

[78] Sitzmann T., Ely K. (2011). A meta-analysis of self-regulated learning in work-related training and educational attainment: What we know and where we need to go. Psychol. Bull..

[79] Stajkovic A.D., Lee D.S. A meta-analysis of the relationship between collective efficacy and group performance. Proceedings of the National Academy of Management meeting.

[80] Kornilov S., Krasnov E., Kornilova T., Chumakova M. (2015). Individual Differences in Performance on Iowa Gambling Task are Predicted by Tolerance and Intolerance for Uncertainty. EAPCogSci.

[81] Furnham A., Marks J. (2013). Tolerance of ambiguity: A review of the recent literature. Psychology.

[82] Chong V.K. (1998). Testing the contingency «fit» between management accounting systems and managerial performance: A research note on the moderating role of tolerance for ambiguity. Br. Account. Rev..

[83] Chatterjee N., Das N. (2015). Key psychological factors as predictors of entrepreneurial success: A conceptual framework. Acad. Entrep. J..

[84] Park J., Chung S., An H., Park S., Lee C., Yoon S.K., Kim S.Y., Lee J.D., Kim K.S. (2012). A structural model of stress, motivation, and academic performance in medical students. Psychiatry Investig..

[85] Sawatzky R.G., Ratner P.A., Richardson C.G., Washburn C., Sudmant W., Mirwaldt P. (2012). Stress and depression in students: The mediating role of stress management self-efficacy. Nurs. Res..

[86] Bashir U., Ismail R.M. (2010). Impact of stress on employees job performance: A study on banking sector of Pakistan. Int. J. Mark. Stud..

[87] Beehr T.A., Jex S.M., Stacy B.A., Murray M.A. (2000). Work stressors and coworker support as predictors of individual strain and job performance. J. Organ. Behav..

[88] Cafagna D., Barattucci M. (2019). Risk perception and personality: A study in the transportation sector. G. Ital. Med. Lav. Ergon..

[89] Hunter L.W., Thatcher S.M.B. (2007). Feeling the heat: Effects of stress, commitment, and job experience on job performance. Acad. Manag. J..

[90] Fredrickson B.L. (2001). The role of positive emotions in positive psychology: The broaden-and-build theory of positive emotions. Am. Psychol..

[91] Wright T.A., Cropanzano R., Bonett D.G. (2007). The moderating role of employee positive wellbeing on the relation between job satisfaction and job performance. J. Occup. Health Psychol..

[92] Dhir R., Sharma V. (2020). Exploring dimensions of psychological capital through grounded theory investigations. Int. J. Indian Cult. Bus. Manag..

[93] Magnano P., Santisi G., Platania S., Zammitti A., Tous Pallares J. (2020). The Italian version of the Work Psychosocial Climate Scale (Escala Clima Psicosocial en el Trabajo). WORK A J. Prev. Assess. Rehabil..

[94] Paolillo A., Pasini M., Silva S.A., Magnano P. (2017). Psychometric properties of the Italian adaptation of the Mor Barak et al. Diversity climate scale. Qual. Quant..

